# Sex Differences in the Use of Complementary and Alternative Medicine among Adults with Multiple Chronic Conditions

**DOI:** 10.1155/2016/2067095

**Published:** 2016-04-27

**Authors:** Monira Alwhaibi, Usha Sambamoorthi

**Affiliations:** ^1^Department of Pharmaceutical Systems and Policy, School of Pharmacy, West Virginia University, P.O. Box 9510, Morgantown, WV 26506-9510, USA; ^2^Department of Clinical Pharmacy, School of Pharmacy, King Saud University, P.O. Box 2454, Riyadh 11451, Saudi Arabia

## Abstract

*Objective*. To examine sex differences in complementary and alternative medicine (CAM) use among adults with multiple chronic conditions.* Methods*. This study used a cross-sectional design with data from the 2012 National Health Interview Survey. The participants were interviewed in 2012 and the reference period for the questions in the survey varied from 1 week to 12 months prior to the interview date. The study included adults (age > 21 years) with no missing data on CAM use variables and who had multiple chronic conditions. Multivariable regression analyses were used to examine the association between sex and CAM use.* Results*. A significantly higher percentage of women compared to men had ever used CAM (51.5% versus 44.3%); women were more likely to have ever used CAM (AOR = 1.49, 95% CI = 1.35–1.65). Among CAM users, a higher percentage of women compared to men used CAM in the past 12 months (53.5% vs. 42.7%); women were more likely to use CAM in the past 12 months (AOR = 1.71, 95% CI = 1.49–1.97). Factors associated with CAM use in the past 12 months were different for men and women; income and obesity were associated with CAM use in the past 12 months among women and not among men.* Conclusion*. Among adults with multiple chronic conditions, women were more likely to use CAM as compared to men.

## 1. Introduction

Complementary and alternative medicine (CAM) use is highly prevalent among adults. The worldwide prevalence of CAM use among adults can be as high as 75% [1]. In the United States (US), approximately 38% of adults use CAM [2]. Generally, adults use CAM to relieve negative symptoms of illnesses and to improve overall health [3]; CAM is also used because individuals experience side effects and do not get relief from conventional therapy [4]. CAM use is influenced by many factors including sex [5–7]. In adults with and without chronic conditions, sex differences in CAM use exist [5–10]. For example, among adults with diabetes women were more likely to use CAM than men [9, 10]. Using data from the 2012 National Health Interview Survey (NHIS), one study reported that among adults with multiple chronic conditions (MCCs) a higher percentage of women used CAM as compared to men (52% vs. 44%) [5].

While the abovementioned studies documented higher rates of CAM use among women as compared to men, they did not examine whether there are sex differences in the association between demographic characteristics, socioeconomic characteristics, health status, and personal health practices and CAM use. Identifying the factors that influence the CAM use among men and women with MCCs is important because of the high and increasing prevalence of MCCs in the United States (US), specifically among women. It is estimated that one in four individuals has MCCs in all age groups, and the majority of those with MCCs are women [11]. Therefore, the objectives of the current study are to (1) examine sex differences in the use of CAM among adults with MCCs and (2) analyze sex differences in the associations between demographic characteristics, socioeconomic characteristics, health status, and personal health practices and CAM use among adults with MCCs.

## 2. Methods

### 2.1. Study Design

This study was a retrospective cross-sectional study using the National Health Interview Survey (NHIS) for the year 2012 among adults with multiple chronic conditions. Multiple chronic conditions were defined as having two or more chronic physical or mental health conditions.

### 2.2. Data Source

The current study used data from an annual survey of households in the US, the 2012 NHIS. The NHIS contains both core (household composition, family, sample child, and sample adults) and supplemental files. A randomly selected adult member (≥18 years old) of a household was invited to complete the Adult CAM use questions, which resulted in the creation of adult CAM supplement file. The core files provide information on demographic characteristics, socioeconomic variables, chronic physical and mental health conditions, functional status, health status, and other variables. Chronic conditions were elicited by asking the participants whether they have ever been told by a doctor or other health professionals that they had a chronic condition. The list of chronic conditions included asthma, arthritis, cancer, chronic obstructive pulmonary disease (COPD), diabetes, heart diseases (angina pectoris, coronary heart disease, heart attack, stroke, and other heart conditions), hyperlipidemia, and hypertension, bipolar disorder, depression, or other mental health disorders. The CAM supplement file was used to obtain information on whether the respondent ever used CAM (see Tables 4 and 5) and, if so, whether they used CAM in the past 12 months. In this study, CAM use was identified using responses to questions related to 18 types of CAM.

### 2.3. Study Sample

The study sample comprised adults, aged >21 years, who responded to both adult core file and the CAM supplementary file. The study sample was further restricted to adults who reported having MCCs (i.e., two or more chronic physical conditions or having a combination of chronic physical and mental health conditions). MCCs were defined from the following list of chronic physical and mental health conditions: asthma, arthritis, cancer, chronic obstructive pulmonary disease (COPD), diabetes, heart diseases, hyperlipidemia, hypertension, bipolar disorder, depression, or other mental health disorders. In the study sample, we only included adults without any missing data on variables related to CAM use. The study sample consisted of 13,246 participants.

### 2.4. Measures

#### 2.4.1. Dependent Variables:* Ever Used CAM*

Adults who have ever used any of the following types of CAM (acupuncture, ayurveda, homeopathy, naturopathy, alexander technique, chiropractic or osteopathic manipulation, craniosacral therapy, feldenkrais, massage, pilates, trager psychophysical integration, biofeedback, chelation therapy, energy healing therapy, hypnosis, qi gong, tai chi, and yoga) were defined as CAM users. Adults who used none of the 18 types of CAM were considered as nonusers of CAM.

*CAM Use in the Past 12 Months among CAM Users*. Adults who never used CAM were excluded and CAM use in the past 12 months was categorized into two groups among CAM users: (1) CAM users in the past 12 months; (2) nonusers of CAM in the past 12 months. Adults who reported using, at least, one of the 18 CAM types in the past 12 months were considered as “CAM users in the past 12 months.” Adults who used none of the 18 types of CAM in the past 12 months were considered as “nonusers of CAM in the past 12 months.”

#### 2.4.2. Independent Variables

Variables used to examine the factors associated with CAM use in both men and women included demographic characteristics (age group in years, race/ethnicity, marital status, and the region of residence), socioeconomic characteristics (education, poverty status as measured by the federal poverty level (FPL), and health insurance coverage), health status (perceived health status, functional limitations, and the type of MCCs), and personal health practices (body mass index, smoking status, alcohol use, and physical activity (i.e., exercise)).

### 2.5. Statistical Analyses

Chi-square tests were used to describe sex differences in demographic characteristics, socioeconomic characteristics, health status, and personal health practices and to examine sex differences in CAM use. The adjusted relationships between sex and ever used CAM and CAM use in the last 12 months were examined with logistic regression models in which independent variables were entered in blocks. We included all the independent variables in the bivariate analyses in the multivariate analysis regardless of significance. Model I examined the unadjusted association between sex and CAM use (i.e., without controlling for any other independent variables). Model II controlled for demographic characteristics (age, race/ethnicity, and the region of residence). Model III controlled for socioeconomic characteristics (education level, marital status, health coverage, and poverty status). Model IV controlled for health status (perceived health, functional limitations, and MCCs) and Model V controlled for personal health practices (body mass index, smoking status, alcohol use, and physical activity).

In order to examine sex differences in the association between demographic characteristics, socioeconomic characteristics, health status, and personal health practices and ever used CAM and CAM use in the past 12 months, separate logistic regressions among women and men were conducted. All the analyses controlled for the complex survey design of NHIS and were conducted using survey procedure with Statistical Analysis System Software (SAS 9.4 Institute Inc., Cary, NC, USA).

## 3. Results

### 3.1. Description of the Study Sample by Sex

Table 1 displays the characteristics of the total study sample (*N* = 13,246) and the characteristics of the study sample by sex. There were statistically significant differences between men and women in demographic characteristics, socioeconomic characteristics, health status, and personal health practices. For example, as compared to men, a significantly higher percentage of women were poor (10.2% versus 14.2%). Also, as compared to men, a significantly higher percentage of women had combined physical and mental MCCs (37.8% versus 24.4%).

### 3.2. Sex Differences in Ever Used CAM

Table 2 summarizes unadjusted differences in CAM use by sex. There were statistically significant differences in CAM use between women and men. As compared to men, a significantly higher percentage of women had ever used CAM (44.3% versus 51.9%). The logistic regressions on CAM use revealed that women were more likely than men to have ever used CAM. The Odds Ratios (OR) and 95% confidence intervals (CI) for women after adjustment for demographic characteristics, socioeconomic status, health status, and personal health practices were as follows: OR = 1.33, 95% CI = 1.22, 1.44 in Model 1 and adjusted OR (AOR) = 1.49, 95% CI = 1.35, 1.65 in Model 4 (Table 3).

### 3.3. Sex Differences in CAM Use in the Past 12 Months among CAM Users

Among CAM users, a statistically significant sex difference in CAM use in the past 12 months was observed. As compared to men, a significantly higher percentage of women used CAM in the past 12 months (42.7% versus 53.5%) (Table 2). The logistic regressions on CAM use in the past 12 months revealed that women were more likely than men to use CAM in the past 12 months. The Odds Ratios and 95% confidence intervals for women after adjustment for demographic characteristics, socioeconomic status, health status, and personal health practices were as follows: OR = 1.56, 95% CI = 1.36, 1.78 in Model 1 and AOR = 1.62, 95% CI = 1.41, 1.87 in Model 4 (Table 3).

### 3.4. Sex Differences in Factors Affecting CAM Use

We found that factors associated with CAM use in the past 12 months were different for men and women. Women with low income were less likely to use CAM in the past 12 months as compared to women with high income (AOR = 0.5, 95% CI = 0.36, 0.73); this was not the case with men. Men with functional limitations were more likely to use CAM in the past 12 months as compared to men with no functional limitations (AOR = 1.1, 95% CI = 1.10, 1.74). However, there was no significant relationship between functional status and CAM use in the past 12 months among women.

## 4. Discussion

This study used data from the 2012 NHIS to evaluate sex differences in CAM use among adults with MCCs and filled knowledge gap about the sex differences in factors affecting CAM use. Past studies in the literature have investigated sex differences in CAM use among adults with and without a specific condition (e.g., diabetes versus no diabetes) [5–8, 10]. To date, the current study is the first one to examine sex differences in CAM use among adults with MCCs. In the current study, even after adjustment for many factors, sex differences in CAM use persisted. Other studies have attributed the higher rates of CAM use among women as compared to men to the sex differences in propensity to seek care [12, 13]. Future research needs to explore factors such as attitude, preferences, and failure of conventional therapies to relieve suffering from chronic illnesses to explain sex differences in CAM use.

Although many studies have documented that women were more likely to use CAM than men [5–8, 10], our study extended the prior literature by also evaluating the factors associated with CAM use in women and men separately. We found sex differences in factors that affect CAM therapy in the past 12 months. For example, women with lower income levels were less likely to use CAM in the past 12 months as compared to those with high income. This was not the case among men. We also found that 33% of women and 37% of men had high income as measured by FPL. These findings suggest that low income may be greater barrier to CAM use among women than among men. Future research needs to explore whether policies that reduce economic disparities between men and women can eliminate the sex disparities in CAM use due to income.

Women with obesity were less likely to use CAM in the past 12 months as compared to women with normal BMI; this was not the case with men. Although prior studies have documented lower rates of CAM use among adults with obesity [14], they did not analyze the interaction between sex and obesity. While we do not know the reasons for this finding, there is some evidence that women with obesity may avoid healthcare services [15]. Future research needs to explore the barriers to CAM use among women with obesity.

Women with both physical and mental health conditions were more likely to use CAM in the past 12 months as compared to those women with only chronic physical conditions; no such relationship was found among men. We also found that a higher percentage of women (37.8%) than men (24.4%) suffered from both chronic physical and mental health conditions. In prior studies, it has been found that a higher percentage of women than men used CAM therapies for mental health conditions [16]. Given that many women suffer from depression and depression was included in our list of MCCs, the higher prevalence of CAM use among women may also be driven by the presence of depression among women. Previous studies have found that women with depression use therapies such as chiropractic, massage, and acupressure to relieve depressive symptoms [4]. Therefore, it is plausible that the type of MCCs was related to CAM use among women and not among men. We observed that men with functional limitations were more likely to use CAM as compared to those without functional limitations; this relationship was not found among women. This was unexpected as prior research among documented women with functional limitations had a higher use of CAM as compared to those without functional limitations [17]. However, these studies were not specific to individuals with MCCs. It is plausible that among women and men with MCCs functional limitation may not be a barrier to CAM use. Future research needs to explore this issue further.

This study has many advantages. It used a nationally representative data with large sample size and included adults with MCCs. It also evaluated the association between sex and CAM use after controlling for a comprehensive list of factors that affect CAM use. However, results of this study should be interpreted in the context of some limitations. All measures in the study were self-reported and thus subject to recall bias. This study did not control for other factors that affect the use of CAM such as severity of chronic conditions, pain, fatigue, beliefs, and attitudes towards CAM use.

## 5. Conclusion

The current study sought to understand the sex differences in the use of CAM among adults with MCCs. We found that women were more likely to use CAM as compared to men and factors affecting CAM use in the past 12 months were different for women and men. As the clinical efficacy and effectiveness of many of the CAM therapies for treating chronic conditions have not been established, healthcare providers treating women for chronic conditions need to be aware of the high prevalence of CAM use among women with MCCs.

## Figures and Tables

**Table 1:** Description of the study sample by sex. National Health Interview Survey 2012.

	Total sample		Women		Men		Sig
	*N*	Wt%	*N*	Wt%	*N*	Wt%	
All	13,246	100	7,738	54.4	5,508	45.6	
Age in years							*∗∗∗*
22–39 years	1,545	12.1	966	12.5	579	11.6	
40–49 years	1,711	14.9	956	14.0	755	15.9	
50–64 years	4,658	36.9	2,603	35.8	2,055	38.2	
65 and older	5,332	36.1	3,213	37.7	2,119	34.2	
Race/ethnicity							
White	8,941	75.1	5,182	74.8	3,759	75.4	
African American	2,125	11.3	1,311	11.7	814	10.7	
Latino	1,476	9.3	870	9.5	606	9.1	
Other race	704	4.3	375	3.9	329	4.8	
Marital status							*∗∗∗*
Married	6,346	63.1	3,162	56.0	3,184	71.6	
Widow/sep/div	5,154	26.9	3,577	34.4	1,577	18.0	
Never married	1,724	10.0	986	9.6	738	10.4	
Education level							
LT high school	2,421	15.6	1,453	16.5	968	14.6	
High school	3,709	28.1	2,163	28.5	1,546	27.7	
GT high school	7,067	56.2	4,095	55.0	2,972	57.7	
Poverty status							*∗∗∗*
Poor	2,239	12.4	1,494	14.2	745	10.2	
Near poor	2,565	16.4	1,589	17.7	976	14.9	
Middle income	3,321	26.0	1,882	26.0	1,439	26.0	
High income	3,588	33.0	1,829	29.1	1,759	37.7	
Insurance							
Insured	11,869	90.1	6,955	90.1	4,914	90.0	
Uninsured	1,352	9.9	769	9.9	583	10.0	
Perceived health status							*∗*
Excellent	1,409	11.6	795	10.8	614	12.6	
Very good	3,351	27.5	1,961	27.7	1,390	27.2	
Good	4,628	34.6	2,694	34.4	1,934	35.0	
Fair	2,801	19.0	1,638	19.3	1,163	18.7	
Poor	1,050	7.3	646	7.9	404	6.6	
Functional limitation							*∗∗∗*
Yes	8,535	62.0	5,343	67.6	3,192	55.3	
No limitation	4,700	38.0	2,388	32.4	2,312	44.7	
MCCs							*∗∗∗*
Physical and mental	4,370	31.7	2,967	37.8	1,403	24.4	
Physical only	8,876	68.3	4,771	62.2	4,105	75.6	
Body Mass Index							*∗∗∗*
Under weight	180	1.2	143	1.7	37	0.5	
Normal weight	3,204	23.1	2,053	26.1	1,151	19.5	
Over weight	4,521	35.1	2,266	29.7	2,255	41.5	
Obese	4,957	37.5	2,936	37.2	2,021	37.9	
Smoking status							*∗∗∗*
Never smoked	6,473	48.4	4,283	54.9	2,190	40.6	
Past smoker	4,178	33.0	2,048	27.5	2,130	39.7	
Current smoker	2,575	18.6	1,395	17.7	1,180	19.7	
Alcohol use							*∗∗∗*
Light/abstainer	2,795	18.7	2,098	24.8	697	11.5	
Former drinker	5,004	37.2	3,028	39.7	1,976	34.2	
Current drinker	5,343	43.4	2,557	34.9	2,786	53.5	
Physical activity							*∗∗∗*
Daily	735	5.4	356	4.5	379	6.5	
Weekly	2,970	24.8	1,562	22.1	1,408	28.0	
Monthly/yearly	8,868	65.2	5,399	68.6	3,469	61.1	
Unable	590	4.0	377	4.3	213	3.6	
Region							
Northeast	2,193	17.2	1,308	17.5	885	16.8	
Midwest	2,814	23.4	1,643	23.6	1,171	23.2	
South	5,021	38.2	2,971	38.4	2,050	37.9	
West	3,218	21.3	1,816	20.6	1,402	22.1	

Note: based on 13,246 adults, age over 21 years, who had two or more chronic conditions (asthma, arthritis, cancer, chronic obstructive pulmonary disease, diabetes, heart diseases, hyperlipidemia, hypertension, bipolar disorder, depression, or other mental health disorders). Asterisks represent significant sex differences in baseline characteristics based on chi-square tests.

Missing indicators for alcohol use, exercise, body mass index, and poverty status were used but are not presented in the table.

GT: greater than; LT: less than; Wt: weighted; MCCs: multiple chronic conditions; Wid/div/sep: widowed, divorced, and separated.

^*∗∗∗*^ *p* < .001; ^*∗*^.01 ≤ *p* < .05.

**Table 2:** Number and weighted percent of complementary and alternative medicine use by sex. National Health Interview Survey 2012.

	Total sample		Women		Men		Sig
	*N*	Wt%	*N*	Wt%	*N*	Wt%	
Ever used CAM (*N* = 13,246)							*∗∗∗*
Yes	6,212	48.2	3,839	51.5	2,373	44.3	
No	7,034	51.8	3,899	48.5	3,135	55.7	
All	13,246	100	7,738	100	5,508	100	
CAM use, past 12 months (*N* = 6,212)							*∗∗∗*
Yes	3,037	49.0	2,015	53.5	1,022	42.7	
No	3,175	51.0	1,824	46.5	1,351	57.3	
All	6,212	100	3,839	100	2,373	100	

Note: based on adults, age over 21 years, who had two or more chronic conditions (asthma, arthritis, cancer, chronic obstructive pulmonary disease, diabetes, heart diseases, hyperlipidemia, hypertension, bipolar disorder, depression, or other mental health disorders).

CAM use was based on 13,246 adults, and CAM use in the past 12 months was based on 6,212 CAM users.

Asterisks represent significant sex differences by Complementary Alternative Medicine use based on chi-square tests.

CAM: Complementary Alternative Medicine; Wt: weighted.

^*∗∗∗*^ *p* < .001.

**Table 3:** Adjusted odds ratios and 95% confidence intervals of women from pooled logistic regressions on ever used CAM and CAM use in the past 12 Months among CAM users. National Health Interview Survey 2012.

	Ever used CAM (*N* = 13,246)			CAM use in past 12 months (*N* = 6,212)		
	AOR	95% CI	sig	AOR	95% CI	sig
Model I, adjusted for only sex						
Women	1.33	[1.22,1.44]	*∗∗∗*	1.56	[1.36,1.78]	*∗∗∗*
Men (ref)						

Model II, adjusted for demographic characteristics						
Women	1.43	[1.31,1.55]	*∗∗∗*	1.61	[1.40,1.85]	*∗∗∗*
Men (ref)						

Model III, adjusted for demographic characteristics and socioeconomic characteristics						
Women	1.48	[1.35,1.62]	*∗∗∗*	1.63	[1.42,1.87]	*∗∗∗*
Men (ref)						

Model IV, adjusted for demographic characteristics, socioeconomic characteristics, and health status						
Women	1.34	[1.22,1.48]	*∗∗∗*	1.56	[1.36,1.79]	*∗∗∗*
Men (ref)						

Model V, adjusted for demographic characteristics, socioeconomic characteristics, health status, and personal health practices						
Women	1.49	[1.35,1.65]	*∗∗∗*	1.62	[1.41,1.87]	*∗∗∗*
Men (ref)						

Note: logistic regression on CAM use was based on 13,246 adults, age over 21 years, who had two or more chronic conditions (asthma, arthritis, cancer, chronic obstructive pulmonary disease, diabetes, heart diseases, hyperlipidemia, hypertension, bipolar disorder, depression, or other mental health disorders). Logistic regression on CAM use in past 12 months was based on 6,212 CAM users. Asterisks represent significant sex differences based on logistic regressions on CAM use and CAM use in the past 12 months.

AOR: adjusted odds ratios; CAM: Complementary Alternative Medicine; CI: confidence interval; ref: reference group.

^*∗∗∗*^ *p* < .001.

**Table 4:** Adjusted odds ratios and 95% confidence intervals of significant independent variables from separate logistic regressions of women and men on ever used CAM. National Health Interview Survey 2012.

	Women			Men		
	AOR	95% CI	Sig	AOR	95% CI	Sig
Race/ethnicity						
White (ref)						
African American	0.57	[0.47,0.69]	*∗∗∗*	0.54	[0.43,0.68]	*∗∗∗*
Latino	0.68	[0.55,0.84]	*∗∗∗*	0.68	[0.51,0.89]	*∗∗*
Other	0.96	[0.70,1.31]		0.74	[0.52,1.04]	
Marital status						
Married	0.97	[0.79,1.18]		1.50	[1.18,1.91]	*∗∗∗*
Widow/sep/div	0.97	[0.81,1.17]		1.33	[1.02,1.73]	*∗*
Never married (ref.)						
Education level						
LT high school	0.46	[0.38,0.56]	*∗∗∗*	0.52	[0.41,0.65]	*∗∗∗*
High school	0.58	[0.50,0.68]	*∗∗∗*	0.84	[0.72,0.99]	*∗*
GT high school (ref)						
Poverty status (FPL)						
Poor	0.44	[0.34,0.56]	*∗∗∗*	0.68	[0.48,0.96]	*∗*
Near poor	0.74	[0.60,0.91]	*∗∗*	0.65	[0.51,0.84]	*∗∗∗*
Middle income	0.70	[0.58,0.85]	*∗∗∗*	0.90	[0.75,1.08]	
High Income (ref)						
MCCs						
Physical only (ref)						
Physical and mental	1.78	[1.54,2.06]	*∗∗∗*	1.57	[1.29,1.91]	*∗∗∗*
Functional limitation						
Limitation	1.61	[1.37,1.90]	*∗∗∗*	1.69	[1.42,2.01]	*∗∗∗*
No limitation (ref)						
Smoking status						
Never smoked (ref)						
Past smoker	1.11	[0.95,1.31]		1.18	[1.00,1.41]	
Current smoker	0.78	[0.66,0.93]	*∗∗*	1.01	[0.81,1.26]	
Alcohol use						
Lifetime abstainer (ref)						
Former drinker	1.40	[1.18,1.65]	*∗∗∗*	1.49	[1.18,1.87]	*∗∗∗*
Current drinker	1.80	[1.49,2.17]	*∗∗∗*	1.49	[1.18,1.89]	*∗∗∗*
Physical activity						
Weekly (ref)						
Daily	1.02	[0.71,1.46]		1.07	[0.80,1.45]	
Monthly/yearly	0.56	[0.47,0.68]	*∗∗∗*	0.79	[0.67,0.92]	*∗∗*
Unable	0.41	[0.28,0.62]	*∗∗∗*	0.54	[0.36,0.83]	*∗∗*
Region						
Northeast	0.56	[0.45,0.69]	*∗∗∗*	0.67	[0.52,0.86]	*∗∗*
Midwest	0.87	[0.71,1.07]		0.73	[0.58,0.92]	*∗∗*
South	0.53	[0.44,0.64]	*∗∗∗*	0.58	[0.48,0.71]	*∗∗∗*
West (ref)						

Note: based on 13,246 adults, age over 21 years, who had two or more chronic conditions (asthma, arthritis, cancer, chronic obstructive pulmonary disease, diabetes, heart diseases, hyperlipidemia, hypertension, bipolar disorder, depression, and other mental health disorders). Asterisks represent significant group differences compared to the reference group based on binary logistic regressions on ever used CAM.

AORs for the following variables are not presented because they were not statistically significant: age, health insurance coverage, and perceived health status.

Missing indicators for alcohol use, exercise, body mass index, and poverty status were used but are not presented in the table.

AOR: adjusted odds ratios; CI: confidence interval; LT: less than; GT: greater than; MCCs: multiple chronic conditions; Wid/div/sep: widowed, divorced, and separated; Wt: weighted.

^*∗∗∗*^ *p* < .001; ^*∗∗*^.001 ≤ *p* < .01; ^*∗*^.01 ≤ *p* < .05.

**Table 5:** Adjusted odds ratios and 95% confidence intervals of significant independent variables from logistic regression on complementary and alternative medicine use in the past 12 months among adults with complementary and alternative medicine use. National Health Interview Survey 2012.

	Women			Men		
	AOR	95% CI	Sig	AOR	95% CI	Sig
Age group						
22–39 (ref)						
40–49	0.72	[0.51,1.00]		0.58	[0.37,0.89]	*∗∗*
50–64	0.55	[0.41,0.73]	*∗∗∗*	0.49	[0.33,0.70]	*∗∗∗*
65+	0.41	[0.30,0.57]	*∗∗∗*	0.39	[0.25,0.59]	*∗∗∗*
Education						
LT high school	0.58	[0.43,0.79]	*∗∗∗*	0.85	[0.57,1.28]	
High school	0.88	[0.70,1.10]		0.71	[0.53,0.93]	*∗*
GT high school (ref)						
Poverty status						
Poor	0.51	[0.36,0.73]	*∗∗∗*	0.72	[0.45,1.17]	
Near poor	0.58	[0.44,0.77]	*∗∗∗*	0.70	[0.47,1.03]	
Middle income	0.64	[0.51,0.81]	*∗∗∗*	0.95	[0.71,1.27]	
High income (ref)						
MCCs						
Physical only (ref)						
Physical and mental	1.42	[1.20,1.69]	*∗∗∗*	1.28	[0.99,1.65]	
Functional limitation						
Limitation	0.90	[0.73,1.11]		1.39	[1.10,1.74]	*∗∗*
No limitation (ref)						
Body Mass Index						
Under weight	0.83	[0.42,1.65]		4.44	[1.13,17.42]	*∗*
Normal weight (ref)						
Over weight	0.91	[0.72,1.15]		1.00	[0.76,1.32]	
Obese	0.78	[0.63,0.97]	*∗*	0.88	[0.64,1.22]	
Smoking status						
Never smoked (ref)						
Past smoker	0.91	[0.73,1.13]		0.92	[0.71,1.20]	
Current smoker	0.69	[0.54,0.88]	*∗∗*	0.68	[0.49,0.94]	*∗*
Physical activity						
Weekly (ref)						
Daily	0.94	[0.62,1.44]		1.15	[0.74,1.78]	
monthly/yearly	0.69	[0.56,0.85]	*∗∗∗*	0.65	[0.50,0.84]	*∗∗*
Unable	0.57	[0.34,0.97]	*∗*	1.06	[0.56,1.99]	
Region						
Northeast	1.25	[0.94,1.67]		0.83	[0.60,1.13]	
Midwest	0.97	[0.75,1.25]		0.80	[0.60,1.06]	
South	0.75	[0.59,0.95]	*∗*	0.74	[0.56,0.96]	*∗*
West (ref)						

Note: based on 6,212 CAM users, age over 21 years, who had two or more chronic conditions (asthma, arthritis, cancer, chronic obstructive pulmonary disease, diabetes, heart diseases, hyperlipidemia, hypertension, bipolar disorder, depression, and other mental health disorders). Asterisks represent significant group differences compared to the reference group based on binary logistic regressions on ever used CAM.

AORs for the following variables are not presented because they were not statistically significant: race/ethnicity, health insurance coverage, and perceived health status.

Missing indicators for alcohol use, exercise, body mass index, and poverty status were used but are not presented in the table.

AOR: adjusted odds ratios; CI: confidence interval; LT: less than; GT: greater than; MCCs: multiple chronic conditions; Wid/div/sep: widowed, divorced, and separated; Wt: weighted.

^*∗∗∗*^ *p* < .001; ^*∗∗*^.001 ≤ *p* < .01; ^*∗*^.01 ≤ *p* < .05.
